# Is This a Helpful YouTube Video? A Research-Based Framework for Evaluating and Developing Conceptual Chemistry Instructional Videos

**DOI:** 10.1021/acs.jchemed.4c01085

**Published:** 2025-01-06

**Authors:** Deborah G. Herrington, Ryan. D. Sweeder

**Affiliations:** † Chemistry Department, Grand Valley State University, Allendale, Michigan 49401, United States; ‡ Lyman Briggs College, Michigan State University, East Lansing, Michigan 48825, United States

**Keywords:** First-Year Undergraduate/General, Multimedia-Based Learning, Equilibrium, Learning Theories

## Abstract

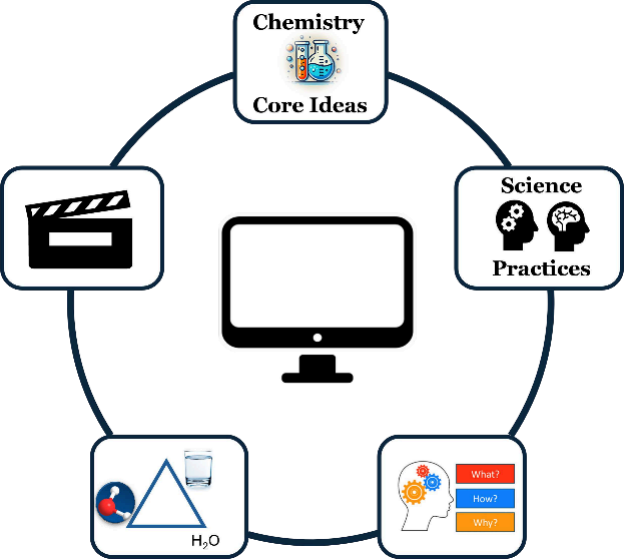

The advent of sites such as YouTube has allowed learners to access videos to support their classroom learning. Given the varying quality and content of chemistry instructional videos, identifying and selecting appropriate videos can be challenging for both instructors and students. This article aims to summarize education research important for creating videos to support students’ conceptual chemistry learning and identify ways these criteria can be operationalized for use in the framework to evaluate or guide the development of instructional videos focused on conceptual understanding of chemistry topics. The framework helps the user consider the chemistry content of the video through the lenses of the disciplinary Core Ideas, Science Practices, causal mechanistic reasoning, and Johnstone’s Triangle. It also includes design considerations from Mayer’s multimedia theory and considerations for accessibility. Finally, we summarize findings and insights gained from using the framework to evaluate a set of 25 highly viewed or highly relevant YouTube videos related to Le Chatelier’s Principle.

The advent of sites like YouTube allows learners to get just-in-time instructional help outside the classroom.^1,2^ This can help meet learners’ unique needs. However, the instructional quality of chemistry videos on these sites varies greatly.^2^ Learners often lack the sophisticated search strategies necessary to find the most salient instructional videos and the prerequisite knowledge to evaluate their quality.^3−6^ Further, the open contribution model for YouTube leads to potentially hundreds of videos addressing a specific topic. Thus, even with adequate prerequisite knowledge, searching for quality videos requires considerable time. Though research has identified instructional features that support students’ conceptual learning of chemistry and important criteria for multimedia learning, these have not been integrated into a research-based framework that can support instructors and researchers in evaluating and developing high-quality chemistry videos. With more people creating videos and more available videos, it is time to start thinking about (1) the quality features of educational videos and (2) how to promote the production of educational videos with these features. This article aims to summarize education research for creating videos to support students’ conceptual chemistry learning, identify ways these criteria can be operationalized for use in a framework to evaluate or guide the development of chemistry instructional videos, and provide examples of the use of this framework for evaluating a set of videos focused on Le Chatelier’s Principle.

## Background

### Use of Videos to Support Learning

Prior to COVID, educational videos had become an important part of education. Learners were increasingly turning to sites like YouTube to support their learning.^1,2,7,8^ One study found that 75% (*N* > 300) of survey respondents from two- and four-year colleges reported actively seeking YouTube videos to learn a biology or chemistry concept.^8^ During the first month of the pandemic people looked to Youtube in unprecedented numbers to support learning, for example, daily views of videos with "homeschool" or "home school" in the title increased over 120% in that time.^9^ With increased use of blended learning and flipped classroom,^10^ the use of chemistry instructional videos will only increase.

However, the quality of YouTube instructional videos varies greatly. Topic-specific studies have shown that few videos contain useful educational information^11^ and a substantial number of videos may contain information opposing scientific consensus views.^12^ Further, studies indicate little or no correlation between the informational or instructional quality of the videos and YouTube’s quality measures (likes, views, and video comments).^12,13^ Additionally, considerations for videos supporting conceptual learning differ from those for videos focused on procedural tasks or skill development. For procedural learning, video choice does not appear to have a large impact, but video choice does have a notable effect on students’ self-motivated learning of concepts.^6^ The plethora of videos, their varying quality, and the lack of correlation between popularity and quality make identifying good videos challenging and time-consuming. Thus, a clear set of criteria to consider when evaluating video quality can help instructors identify videos for their students and can support content creators in the development of higher-quality chemistry instructional videos.

### General Considerations for Video Design and Evaluation

General criteria for the development or evaluation of instructional videos or video explanations have been proposed,^14^ and several studies have examined the efficacy of chemistry instructional videos;^15−20^ however, what is missing is a set of criteria for evaluating the overall instructional quality of conceptual chemistry videos. Although many criteria for effective chemistry teaching in a face-to-face classroom also apply to effective instructional videos, there are also important differences between face-to-face and online learning.^21^ With videos, learners can engage with the content when it is most convenient for them and for as long as needed;^22^ however, learners need to be motivated to effectively engage with this content and understand what they should learn. General criteria for effective instructional videos or video explanations are largely grounded in the cognitive theory of multimedia learning and cognitive load theory.

Mayer and Moreno’s cognitive theory of multimedia learning states that deeper learning can occur when information is presented both verbally and visually.^23^ This theory is built on three assumptions: (1) dual-channel theory—learning is enhanced by simultaneously targeting people’s separate visual and auditory reception channel; (2) limited capacity assumption—people can process a small amount of information, 5–7 chunks, at once; and (3) active processing assumption—learning requires active engagement in cognitive processes (e.g., identifying and selecting relevant information or integrating new models with prior knowledge). The limited capacity and active processing assumptions are related to cognitive load theory^24^ and the ICAP (Interactive, Constructive, Active, and Passive) framework, respectively.^25^

Cognitive load is important in developing conceptual videos, as simultaneous visuals and sound can exceed a learner’s cognitive processing capacity. Accordingly, Mayer identified 12 principles for multimedia learning to reduce extraneous load (cognitive effort wasted on things not supporting learning), manage intrinsic load (cognitive effort required to represent things in working memory), and optimize germane load (effort required to understand the material).^26^

- Multimedia: videos should include narration and visuals
- Coherence: exclude unnecessary information, graphics, and sounds
- Signaling: key points should be highlighted
- Redundancy: graphics or text should complement a spoken presentation
- Spatial contiguity: related text and visuals should be on-screen close together
- Temporal contiguity: narration and related visuals should be presented simultaneously
- Segmenting: content should be organized in manageable, coherent chunks
- Pretraining: ensure that learners have essential prior knowledge of key concepts and terms
- Modality: written text should be limited; rely on visuals and spoken words
- Voice: human voices are better than machine voices
- Personalization: use a conversational tone and first person
- Image: minimize talking heads on the video

These Principles, along with student engagement, serve as the foundation for several video evaluation studies and video analysis frameworks which are summarized in a paper by Ring and Brahm.^14^ Student engagement is inherent in the active processing assumption that underlies the cognitive theory of multimedia learning and is supported by the ICAP hypothesis which postulates that greater engagement with learning materials leads to increased learning.^25^

Other criteria commonly used in video evaluation studies or video analysis frameworks center on the quality of the instructional explanations. In synthesizing several such studies, Ring and Brahm present five criteria categories for evaluating explanation quality, which partially overlap with Mayer’s Principles.

- Content: should be correct, accurate, and complete
- Learner orientation: explanations should be targeted, consider the learner’s prior knowledge, and connect to other knowledge or experiences
- Representations: analogies, models, graphs, diagrams, charts, etc., should clearly represent the principle
- Language: language complexity should allow learners to translate between domain language and everyday terms
- Process structure: explanations should be structured with coherent argumentation followed by a summary

Most studies that used these criteria to develop video analysis instruments focused on one content area, including content-specific criteria not easily adapted to other contexts. Conversely, Ring and Brahm developed a more general set of criteria that could be applied to videos over a wide range of topics; yet, that comes with its own challenges. For example, they define technical completeness as follows: “The video explanation is technically complete if no information or subject-specific terms relevant to the topic or the argument are omitted.” This relies on individual evaluators each deciding what information should be included. For chemistry, research has identified content elements that support student learning that can form the basis of chemistry content-specific criteria.

To date, studies evaluating chemistry videos have not used such chemistry content-specific criteria. One study used Mayer’s Principles to evaluate chemistry videos, finding that of the six elements they coded for, only coherence and organization differentiated videos.^15^ Another provided a set of criteria used to peer-review chemistry videos posted to a YouTube channel for Spanish language chemistry instructional videos. However, these criteria include items such as “the author proposes exercises at the end of the lesson” and “the proposed exercises are solved at the end of the video”,^16^ suggesting a skill development focus. Other studies of chemistry instructional videos have not focused on evaluating video features but rather on evaluating student outcome for videos created by the course instructors for specific purposes such as replacing exam review^17^ or discussion sections,^18^ solving specific organic chemistry synthesis problems,^19^ or online tutorials addressing common homework or exam problems.^20^ Thus, there exists a need for a video evaluation framework that addresses both important elements of multimedia learning and elements that are more specific to the learning of chemistry concepts. Fortunately, research has identified specific elements such as three-dimensional instruction, causal mechanistic reasoning, and connecting levels of Johnstone’s Triangle that support conceptual learning in chemistry and can be operationalized for a chemistry video evaluation framework.

### Chemistry Content Considerations for Educational Videos

Developed by a team of practicing scientists, cognitive scientists, science education researchers, and science and policy experts using a rigorous feedback and revision process, *A Framework for K-12 Science Education* (the Framework)^27^ outlines a vision for science education grounded in research on how students learn science best. It advocates for curricula structured as scaffolded progressions for each of three dimensions: disciplinary core ideas (fundamental concepts that underpin a discipline), scientific and engineering practices (how scientists construct and use knowledge), and crosscutting concepts (tools or lenses used across disciplines for making sense of phenomena).^27−31^ Such three-dimensional learning (3DL) promotes the development and use of interconnected knowledge that is more expert-like in nature,^27−29,31−34^ in contrast to more traditional science instruction and assessment that treats science as a collection of facts and skills,^35,36^ frequently resulting in fragmented learning.^28,29,33,37^ The 3DL approach actively engages learners in the process of science such as making predictions and constructing scientific explanations about phenomena. In chemistry, an important outcome of 3DL is the ability to explain the macroscopic properties of materials and phenomena within and beyond the discipline.^38^ This causal mechanistic reasoning (CMR) requires explanation at a level below that of the phenomenon of interest.^39^ For chemistry, this generally involves the use of atomic or molecular level motion and interactions to explain observable phenomena.^40^

The ability to meaningfully connect macroscopic observations with particle behavior is a challenge for novice chemistry learners, recognized in Alex Johnstone’s seminal work.^41^ He noted that a deep, conceptual understanding of chemistry requires integration of knowledge on three levels: (1) macroscopic—observable by the senses; (2) particulate—interactions and movements of atoms, ions, and molecules; and (3) symbolic—representations of macroscopic and particulate using symbols, formulas, equations, mathematical relationships, and graphs. What makes chemistry challenging for novice learners is connecting these three levels.^42−46^ If not given appropriate time and opportunity to integrate these levels of representation, learners build fragmented mental models of concepts.^47^

## Framework for Video Evaluation

A valuable framework for the evaluation and development of quality chemistry instructional videos to support conceptual learning should incorporate both aspects unique to the teaching and learning of chemistry content and elements that are important for multimedia learning. We propose that the evaluation criteria providing the basis of this framework should be informed by 3DL, CMR, Johnstone’s Triangle, active engagement and the ICAP framework, and the cognitive theory of multimedia learning. In the following sections, we describe each criterion in detail, outline how these criteria can be operationalized, and provide a summary of findings in applying these criteria to 25 highly viewed/most relevant Le Chatelier’s Principle (LCP) YouTube videos. A complete list of videos, coding results, and exemplars are included in the Supporting Information (SI 1 and SI 2). General video selection criteria included the following: over 100,000 views or highly relevant, in English, under 15 min, and appearing in one of a variety of related searches.^15^

### 3DLearning: Core Ideas

Chemistry Core Ideas are explanatory and generative concepts fundamental to chemistry that underlie the typically taught general chemistry topics. The Framework defines the Disciplinary Core Ideas for K-12 levels. Core Ideas central to chemistry are found in PS1: Matter and Its Interactions and PS3: Energy.^27^ At the university level, a set of chemistry Core Ideas (Table 1) were identified by the 3D-LAP (Learning Assessment Protocol) research team and their chemistry colleagues.^29,48^ These Core Ideas differ somewhat from those in the Framework, though they overlap significantly.

**Table 1:** Chemistry Disciplinary Core Ideas from 3D-LAP^29^ or Adapted from the Framework^27^ with Operationalization for LCP

Core Idea	Description	Operationalized for LCP
Energy: macroscopic, atomic/molecular, quantum mechanical	Kinetic and potential energy changes occur when atoms and molecules interact. Energy is released to the surroundings when attractive noncovalent interactions form, and conversely, energy is required to overcome noncovalent interactions.	Changing the temperature causes a shift in an equilibrium by altering the number of collisions that are “successful” in overcoming the activation energy barrier for the reaction. Due to the differences in the activation energy barriers for the forward and reverse reactions, the two processes will be differentially impacted, leading to change in the relative concentrations of the reactants and products.
Change and Stability in Chemical Systems	Energy and entropy changes, the rates of competing processes, and the balance between opposing forces govern the fate of chemical systems.	Change: “stressing” an equilibrium system (changing concentrations or temperature) causes changes in relative rates of the forward and reverse reactions.
		Return to stability: the system “shifts to offset the stress” as the equilibrium system returns to a state where the forward and reverse reaction rates are equal.
Particulate Nature of Matter	Matter is composed of particles (atoms, molecules, ions). Qualitative and quantitative observations about matter (e.g., Brownian motion, ratios of reactants and products in chemical reactions) can be explained in terms of the motion, interactions, and rearrangements of particles.	Stresses on the system will alter the rates of the forward and reverse reactions by changing the number of collisions and consequently the number of successful collisions (collisions with enough energy and correct orientation).
Electrostatic and Bonding Interactions	Attractive and repulsive electrostatic forces govern noncovalent and bonding (covalent and ionic) interactions between atoms and molecules. The strength of these forces depends on the magnitude of the charges involved and the distances between them.	-
Atomic/Molecular Structure and Properties	The macroscopic physical and chemical properties of a substance are determined by the three-dimensional structure, the distribution of electron density, and the nature and extent of noncovalent interactions between the particles.	-

Since Core Ideas are explanatory and broadly applicable, most chemistry topics have multiple Core Ideas. Hence, any video focused on developing a strong conceptual understanding of a topic should clearly connect one or more of the Core Ideas to the topic. Such explanations can help learners build a more cohesive understanding of how different chemistry concepts are related. Table 1 provides the general descriptions of each of the four Core Ideas from the 3D-LAP and an additional Core Idea (Particulate Nature of Matter) from the Framework, and it outlines how the Core Ideas of Energy, Change and Stability, and Particulate Nature of Matter are operationalized for the concept of LCP.

Of the 25 LCP YouTube videos, seven addressed the Core Idea Change and Stability, but none addressed Energy or Particulate Nature of Matter. Most videos approached LCP as a heuristic, focusing on predicting equilibrium shifts in response to applied stresses including changes in concentration, temperature, or pressure/volume (for systems involving gases) but not explaining *why* such shifts occurred. Though several videos noted that equilibrium systems are composed of forward and reverse reactions, few discussed the rates of those reactions or how stresses altered the forward and reverse rates differently to cause the observed changes to equilibrium system. This is most evident in addressing effects of temperature where “heat” was treated as a reactant (endothermic) or product (exothermic) and predictions were made based on the addition/removal of reactant or product, rather than addressing how changing thermal energy unequally affects the rate of successful collisions for the forward and reverse reactions. Ignoring the connection between the reaction rates and their relation to equilibrium state necessitates that the only way to “understand” the topic of LCP is through memorization of a heuristic.

### 3DLearning: Science Practices

The Framework identifies eight Science and Engineering Practices that scientists regularly engage in.^27^ Teaching science content through engagement in Science Practices helps learners understand how scientific knowledge is developed and, thus, supports the development of a more coherent and connected understanding of science concepts. Although videos do not inherently engage the learner in activities, they can model the practices. The Science Practices have been operationalized for classroom instruction, with Engaging in Arguments from Evidence and Constructing Explanation combined.^32^ The specific elements for the four most common practices found in chemistry instructional videos are summarized in Table 2. For a video to be considered as containing a Science Practice, it must meet all of the criteria for the practice.

**Table 2:** Select Science Practice and Criteria as Defined by the 3D-LOP^32^

Science Practice	Criteria (All Must Be Present)
SP 6: Constructing Explanations and Engaging in Argument from Evidence	• Instruction presents an event, observation, or phenomenon.
	• Instruction presents or asks instructor/students to make a claim based on the given event, observation, or phenomenon.
	• Instruction has instructor/students provide scientific principles or evidence (data or observations) to support the claim.
	• Instruction has instructor/students provide reasoning about why the scientific principles or evidence support the claim.
SP 2: Developing and Using Models	• Instruction presents an event, observation, or phenomenon for instructor/students to explain or make a prediction about.
	• Instruction presents a representation or asks instructor/students to construct a representation.
	• Instruction has instructor/students explain or make a prediction about the event, observation, or phenomenon.
	• Instruction has instructor/students provide the reasoning that links the representation to their explanation or prediction.
SP4: Analyzing and Interpreting Data	• Instruction presents a scientific question, claim, or hypothesis to be investigated.
	• Instruction provides a representation of data (table, graph, or list of observations) used to answer the question or test the claim or hypothesis.
	• Instruction provides an analysis of the data or asks students to analyze the data.
	• Instruction has instructor/students interpret the results or assess the validity of the conclusions in the context of the scientific question, claim, or hypothesis.
SP 5: Using Mathematics and Computational Thinking	• Instruction presents an event, observation, or phenomenon.
	• Instruction has instructor/students perform a calculation or statistical test, generate a mathematical representation, or demonstrate a relationship between parameters.
	• Instruction has instructor/students give a consequence or an interpretation in words, diagrams, symbols, or graphs of their mathematical results while demonstrating reasoning in the context of the given event, observation, or phenomenon.

Key for identifying whether Science Practices are incorporated into instruction is the presence of an event, phenomena, observation, claim, or question to be explained or investigated. It is quite common for instruction to have the first several elements of a practice but be missing the last criterion practice (providing reasoning or interpretation). The practices most frequently found in the LCP videos were Analyzing and Interpreting Data and Using Mathematics and Computational Thinking. Several videos met the Analyzing and Interpreting Data criteria by (1) asking what would happen when a certain stress was applied (presenting a situation to be investigated), (2) showing what happened when the system was stressed (providing evidence through observations to answer the question), (3) connecting the color change to the direction of equilibrium shift (providing an analysis of the observations), and (4) explaining the results using LCP (interpreting the results). However, most of these videos did not contain a Core Idea or CMR, as they described what was happening but did not explain why it was happening.

### Causal Mechanistic Reasoning

Chemistry allows us to predict and explain macroscopic observations and the properties of materials using particle motions, interactions, and behaviors.^38^ Such explanations, also known as CMR, demonstrate a deep and connected understanding of chemistry concepts. Creating such explanations is challenging for novice learners, and quality conceptual instruction should focus on helping learners develop CMR. CMR can be viewed as answering three distinct questions about a phenomenon: “what?”, “how?”, and “why?”. As such, videos containing CMR focus less on sharing facts or solving algorithmic problems and more on developing a deeper understanding of the topic. Developing such reasoning supports learners in broadly applying their conceptual understanding to explain or predict what happens in new situations or phenomena. Indeed, students can achieve this level of success using carefully constructed curricular materials with suitable question prompts.^49,50^

For LCP, CMR could answer the question of “what happens to a system in equilibrium when more reactant molecules are added?” Beyond simply saying that such a system would “shift right” or “the system would make more products”, a CMR explanation addresses “how” the change came about and “why” this leads to the observed shift. The increase in reactant concentration will result in more collisions between reactant molecules and increase the forward rate of the reaction (how). Since the rates of the forward and reverse reactions are no longer equal, there will be an accumulation of more products and a reduction in the concentration of reactants until a new equilibrium is established between the forward and reverse reaction rates (why). In this explanation, the reasoning for a bulk measurement (concentration) is explained by particulate movement and interactions, which is the level below the phenomenon of interest. For a given topic, there may be multiple “what” questions that could be addressed. For example, an LCP video might similarly answer the question, “what happens to a system in equilibrium when the temperature is increased?”

Exploring LCP YouTube videos with more than 100,000 views, we did not find any that provided this level of explanation. In fact, it was rare that collisions were discussed at all. This is perhaps not surprising since most videos use LCP as a heuristic. In exploring less frequently viewed LCP videos, we found a few videos that included CMR. It is not clear if the disconnect between views and quality/depth of explanation is driven by the typical classroom assessments that do not require how or why explanations or another factor(s).

### Levels of Representation for Johnstone’s Triangle

Johnstone’s Triangle is an insightful articulation for exploring how chemists think.^41^ Johnstone points out that experts move seamlessly between the macroscopic, particulate, and symbolic levels. Novices, however, tend to reason more along the edges of the triangle and need to gain practice and experience moving between these levels. Research has shown that learners taught to translate between these levels were more successful in solving general and organic chemistry problems.^51,52^ Videos with the ability to include images, animations, simulations, and physical demonstrations can capture and represent each of the different levels in ways that are challenging for traditional classroom instruction. The ability to readily incorporate moving images, animations, etc., provide the potential for supporting viewers in the challenging task of developing their own mental models that connect the different levels of representations.^42−46^ Johnstone noted that traditionally, chemistry instruction has been presented at the symbolic level.^41^ However, he advocated for instruction beginning with the macroscopic level, things directly observable so that learners can connect with their own experiences to help ground newly acquired knowledge. In chemistry, demonstrations and laboratory experiments often provide such macroscopic experiences. Videos have the same potential but can add in particulate level simulations or animations to help learners make connections between the macroscopic and particulate levels. Thus, videos may be uniquely situated to support learners in making connections among these three levels of chemistry. Yet, novice learners cannot be expected to make these connections on their own. Explicit connections between these levels must be included.

Though all the videos analyzed included symbolic representations, few contained macroscopic representations, and particulate level representations were even less common (4/25). This is consistent with an overreliance on the use of the symbolic level in teaching chemistry.^53^ Although learning the symbolic level is important since symbols are the language chemists use for communicating and representing chemical concepts,^54^ decoding the symbolic language provides extra knowledge demands for students^41^ when not explicitly connected to the macroscopic observations or particle interactions they are representing.^54^ Developing learners’ abilities to provide causal mechanistic explanations requires them moving between the levels of Johnstone’s Triangle more fluidly,^41^ which is not possible when only focusing on symbolic representations. More positively, when either the particulate or macroscopic level were present, videos usually made clear connections between multiple types of representations.

### Mayer’s Principles of Multimedia Learning

As described previously, Mayer outlines 12 research-based principles for multimedia learning, but recent work by Magnone et al.^15^ suggests that not all these principles are equally discriminating for chemistry YouTube videos. In their evaluation, they focused on six principles: coherence, signaling, spatial contiguity, temporal contiguity, segmenting, and image, noting the greatest variance across videos for coherence (which they combined with image) and organization (an element of signaling). Additionally, we found that the redundancy and multimedia principles were also important distinguishers between videos. Together, these four criteria address Mayer’s three core ways to support learning processing by (1) reducing extraneous processing, (2) managing essential processing, and (3) fostering generative processing. Our operationalization of the four criteria and the way that each supports learning are outlined in Table 3.

**Table 3:** Operationalization of Mayer’s Criteria for Evaluation of Chemistry Videos

Criteria	Operationalization of Criteria	Support for Learning
Text	Text on screen is minimal (ex: only brief bullet points or keywords if text is used)	Reduces extraneous processing
Segmenting	Contains elements that **meaningfully** support student organizing content or identify key ideas (introductory organizer, section heading, summary, guiding questions/topic (for short, focused video only). Key question: could a novice learner clearly understand what they should take away from the video?	Manages essential processing by identifying key information
Coherence	Content is all relevant to the topic and learning (no music, unhelpful animations, graphics, etc.)	Reduces extraneous processing by removing unnecessary distractors
Image	Video almost exclusively involves **meaningful and relevant** images and verbal components AND **images are explained for a novice learner**	Fosters generative processing and helps integrate content with prior knowledge

Most LCP videos we evaluated avoided excessive text and provided meaningful segmenting to help organize the content, often by providing a summary of the content at the end, segmenting the video with meaningful “chapter” titles, or providing a clear overview at the beginning. Videos were less likely to meet the coherence and multimedia principles. Regarding coherence, many videos contain significant asides to the content or had the narrator of the video on-screen for a significant portion of the video, similar to previous reports.^15^ These provide additional cognitive distractions that may hinder a learner’s ability to focus on the conceptual content. However, it is important to recognize that many videos strive to be both educational and entertaining. Asides and the presence of the narrator on screen may increase interest, which is critical when selecting and watching videos is a choice. Yet, when learning a new and complex concept, too many or lengthy asides or a distracting presence can impede the construction of coherent understanding of a concept, creating a potential dilemma for creators. The key reasons that LCP videos did not meet multimedia expectations were the lack of images to support learning or long video segments with just the narrator talking. This was especially common for older videos of someone giving a lecture in front of a whiteboard or with handwritten notes or videos focused on solving LCP problems that showed the chemical equation of the equilibrium system, identified different stresses applied (written text), and then decided what direction the equilibrium would shift.

### Video Content Accuracy and Accessibility

Ring and Brahm included aspects of content accuracy and accessibility in their Content, Learner Orientation, and Representations criteria.^14^ In addition to content accuracy (incorrect content or misleading content), which we considered a baseline criterion, we specifically focused on the use of analogies and video closed captioning in our framework. Analogies can be very powerful teaching tools, especially for things that cannot be directly observed, such as atoms and molecules and their interactions. They can help learners connect the unobservable to something they have prior knowledge or experience with.^55^ However, analogies are only useful if the learner has knowledge of the analogue example. For example, comparing something to a magnet is not helpful for someone who has never seen or used a magnet. This can be minimized in videos by showing the analogy visually instead of just referring to it verbally. Lacking the visual component adds additional cognitive load to those unfamiliar with the referenced process. Thus, our evaluation of analogy use included notes about whether it was presented just verbally or both verbally and visually.

Our framework also assesses one aspect of universal design, Equitable Use in terms of video captioning.^56^ In addition to supporting hearing impaired learners, captioning can help learners watch videos in their non-native language or in learning new technical terms. YouTube automatically captions videos; however, this auto captioning does not include punctuation or capitalization of words. Further, some of the scientific terms used in chemistry videos, e.g., LCP, are frequently auto captioned incorrectly. Thus, our framework includes criteria for evaluating the quality of captioning.

In our analysis of videos, there were a few cases of incorrect content (unbalanced chemical equations or incorrect explanations), but more frequently there was misleading content. For example, six of the 25 videos used a balance with equal amounts on each side as an analogy for an equilibrium system. The system was *stressed* by adding or removing something from one side of the balance, and *equilibrium* was restored when the amounts on each side were again equal. This is misleading, as it suggests that a system is at equilibrium when there are equal *amounts* of reactants and products as opposed to when the forward and reverse reaction rates are equal. Overall, seven of the 25 videos used some form of analogy with three of them having only verbal reference to the analogy. Interestingly, four of the five videos with over 500k views included analogies. Most of the videos we evaluated relied on auto captioning. However, we also noted that many of the videos by popular content creators were accurately captioned.

### Viewer Engagement

The ICAP (Interactive, Constructive, Active, and Passive) hypothesis predicts that learning increases as learners become more engaged with learning materials, from passive up through interactive.^25^ Students are classified as passive if receiving information (e.g., listening to a lecture or reading a text passage without doing anything), active if manipulating information (taking notes, copying a solution, underlining keywords), constructive if generating additional outputs or products (making connections between topics, solving a problem, explaining, paraphrasing), and interactive if dialoguing (debating, discussing, asking, and answering questions). Accordingly, videos that explicitly attempt to move the learner beyond the passive interaction of just watching the video better support learning. Although learners may opt to take notes or pause and reflect on a topic during any video, this is more likely in videos that explicitly prompt learners to engage with the content. In exploring LCP videos, this sort of prompting was not common (4/25 videos). Although a correlational observation for a single topic, it is notable that this is more frequent among the most highly watched videos (three of the top six most viewed).

### Limitations

This framework was constructed to evaluate the potential of videos to support development of *conceptual* understanding of chemistry topics. There are a plethora of chemistry instructional videos focused on skill development (e.g., balancing equations, drawing Lewis structures), which can be beneficial to learners but are not the focus of this work.

Using dichotomous choices for each criterion (present or not/high or low) facilitates use of the framework and improves inter-rater reliability; however, it does not capture gradations of quality for each of the categories. For example, a 10 min video with only 20–30 s focused on a Core Idea, though very different from one that has the Core Idea embedded throughout the full length of the video, would both score as having a Core Idea present. Similarly, some videos scored as low for coherence because they had the narrator on screen for the full time, whereas others had dozens of visually distracting transitions and long asides.

This framework takes a narrow view of engagement, albeit one that is supported by studies that show learners are more likely to watch and comprehend video content when explicitly encouraged to engage though guiding or embedded questions, or when it is incorporated into course assignments or assessments. This suggests that it is not just the video that is important, but also how it is incorporated into instruction.^57^ However, this framework does not address the entertainment aspect of videos. Wit, humor, a conversational and enthusiastic tone, and personal context are all things that leaners have identified as increasing their motivation to watch videos.^58^ Yet, too many side stories or flashy transitions can distract students from the core content.^26^

## Insights Gained from Using the Framework for Video Evaluation

The research-based criteria outlined above provide a useful framework for the evaluation or development of videos designed to support the conceptual understanding of a chemistry topic. Employing this framework for LCP videos provided an effective lens for evaluating videos. We leave the reader with three specific insights gleaned from the use of this framework and how it can influence thinking about evaluating, creating, and using chemistry conceptual videos.

**The framework outlines important aspects for both consumers and creators to consider regarding the content of chemistry conceptual videos.** To facilitate this, we provide an annotated checklist in the Supporting Information (SI 1). Although criteria such as Mayer’s Principles or Science Practices can be used without modification to evaluate videos across many chemistry concepts, Core Ideas, CMR, and even Johnstone’s Triangle criteria require topic-dependent specificity for how these elements can or should be incorporated into a video. This operationalization for each topic takes time, but this advanced planning allows the evaluator or creator to focus on key elements in supporting learners’ conceptual understanding and ensures the video does not approach a topic from a solely algorithmic perspective. Further, when more than one Core Idea or CMR question is identified for a topic, it could provide a coherent way to break up content for video creators to make several short videos on the same topic.

**The framework can help identify gaps in the existing body of available videos.** Although several videos showed demonstrations of equilibrium systems that change colors as they respond to stresses (meeting the expectations of Science Practice Analyzing and Interpreting Data), most did not contain a Core Idea or CMR, as they focused on describing what was happening but not on explaining why this was happening. This treats LCP as an algorithm to be memorized and applied, as opposed to connecting it to collisions between particles. A fundamental explanation of LCP requires talking about particle collisions, which only three videos did. However, it is interesting to note that *none* of the videos that discussed particle collisions and were coded with CMR included particulate representations. Although when macroscopic or particulate representations were present, there was generally an explicit connection between levels, most videos relied solely on symbolic representations. While we anticipate that the strengths and areas for improvement will be dependent on the topic, this illustrates how using this framework identified areas of improvement for LCP videos.

**Instruction should be designed to account for students watching videos to support their learning of a topic.** The framework can help instructors identify quality videos to recommend to their students to support learning outside of the classroom. However, many students still choose to search for their own videos. Therefore, using the framework to identify key areas for improvement for videos on a given topic can help instructors focus their instruction. For example, knowing that most videos treat LCP as an algorithm, instructors can focus classroom time on helping students understand the underlying mechanism based on particle collisions. Or knowing that the balance analogy is frequently used in videos, instructors can explicitly address the issues with that analogy in the classroom.

## Supporting Information Available

SI 1: evaluation information for Le Chatelier’s Principle for instructors, video exemplars, and checklist for video evaluations (PDF, DOCX)
SI 2: video evaluations for Le Chatelier’s Principle videos (XLSX)
