# Supplementary material for: Is This a Helpful YouTube Video? A Research-Based Framework for Evaluating and Developing Conceptual Chemistry Instructional Videos
Source: J Chem Educ. 2025 Jan 6;102(2):621–9. doi: 10.1021/acs.jchemed.4c01085 (PMC11823403; doi:10.1021/acs.jchemed.4c01085)
Supplement: Supplementary file 1 — ed4c01085_si_001.pdf [file ed4c01085_si_001.pdf]

Title: "Is this a Helpful YouTube Video? A Research-Based Framework for Evaluating and Developing Conceptual Chemistry Instructional Videos"

Author(s): Herrington, Deborah[1]*; Sweeder, Ryan[2]

1 Department of Chemistry; Grand Valley State University

2 Lyman Briggs College; Michigan State University

* Corresponding author: herrringd@gvsu.edu; 1 Campus Drive, Allendale, MI 49401 USA

Supplemental information contents

	Page
**Le Chatelier's Principle (LCP) Evaluation Information Page**	3
**Video exemplars**	7
**Checklist for video evaluation or creation**	8
**References**	11

Le Chatelier's Principle (LCP) Evaluation Information Page

Note: Many videos use a balance analogy which has issues but is NOT coded as a science inaccuracy.

Johnstone's Triangle Elements

Element	Examples
Macroscopic	Visible phenomena - most common ones involve things that change color. For example, cobalt chloride equilibrium system (blue to pink) or N_2O_4 to $2NO_2$ system (brown vs. colorless). This can be a video/pictures of the system or a simulation.
Symbolic	Most common is a chemical equation of the equilibrium system, but some videos show a graph of change of concentrations of reactants and products as the reaction proceeds
Particulate	This tends to be the least common element included in LCP videos. Particle representations should show reactants and products going back and forth. Particles sitting on a balance does NOT meet this criterion. It can be a static picture that shows the ratio of reactant:product particles changing due to the stress. Key idea: particle representation **must** be explicitly linked to the explanation or equilibrium/LCP.
Connection between levels	Videos should make explicit connections between two or more levels for this to be coded as present. For example, they could show a particle level diagram of how reactants are converted to products and then the reverse reaction and connect that to how the concentrations are changing on a concentration vs. time graph

Core Ideas

Core Idea Elements		
Change and Stability in Chemical Systems	Energy	Particulate Nature of Matter
**Change:** “Stressing” an equilibrium system (changing concentrations or temperature) causes changes in relative rates of the forward and reverse reactions<br><br>**Return to Stability:** The system “shift to offset the stress” as the equilibrium system returns to a state where the forward and reverse reactions are equal<br><br>Must have both parts	Changing the temperature causes a shift in an equilibrium by altering the number of collisions that are “successful” in overcoming the activation energy barrier for the reaction. Due to the differences in the activation energy barriers for the forward and reverse reactions, the two processes will be differentially impacted leading to change in the relative concentrations of the reactants and products.	Stresses on the system will alter the rates of the forward and reverse reactions by changing the number of collisions and consequently the number of successful collisions (collisions with enough energy and correct orientation).

Scientific Practices

We expect that the most common practices will be:

- SP 2: Developing & Using Models (e.g. using a simulation to show how changing variables affects the equilibrium system)
- SP 4: Analyzing and Interpreting Data (e.g. looking at specific data about equilibrium systems and explaining it)
- SP 5: Using Mathematical & Computational Thinking (e.g. using K & Q to explain changes in equilibrium systems)
- SP 6: Constructing explanations and engaging in argumentation from evidence

**Scientific Practices Criteria (must satisfy all criteria) From Bain, et al, (2020)**
**SP 1: Asking Questions**<br>❏ Instruction presents an event, observation, phenomenon, data, scenario, or model.<br>❏ Instruction has instructor/students *generate* an empirically testable question about the given event, observation, phenomenon, data, scenario, or model. (Question cannot be rhetorical or simply a part of instruction; rather it must be a *process of generating* a testable question.)
**SP 2: Developing and Using Models**<br>❏ Instruction presents an event, observation, or phenomenon for instructor/students to explain or make a prediction about.<br>❏ Instruction presents a representation or asks instructor/students to construct a representation.<br>❏ Instruction has instructor/students explain or make a prediction about the event, observation, or phenomenon.<br>❏ Instruction has instructor/students provide the reasoning that links the representation to their explanation or prediction.

SP 3: Planning Investigations

- ❑ Instruction poses a scientific question, claim, or hypothesis to be investigated.
- ❑ Instruction has instructor/students describe or design an investigation, or identify the observations required to answer the question or test the claim or hypothesis.
- ❑ Instruction has instructor/students justify how their description, design, or observations can be used to answer the question or test the claim or hypothesis.

SP 4: Analyzing and Interpreting Data

- ❑ Instruction presents a scientific question, claim, or hypothesis to be investigated.
- ❑ Instruction provides a representation of data (table, graph, or list of observations) used to answer the question or test the claim or hypothesis.
- ❑ Instruction provides an analysis of the data or asks instructor/students to analyze the data.
- ❑ Instruction has instructor/students interpret the results or assess the validity of the conclusions in the context of the scientific question, claim, or hypothesis.

SP 5: Using Mathematics and Computational Thinking

- ❑ Instruction presents an event, observation, or phenomenon.
- ❑ Instruction has instructor/students perform a calculation or statistical test, generate a mathematical representation, or demonstrate a relationship between parameters.
- ❑ Instruction has instructor/students give a consequence or an interpretation in words, diagrams, symbols, or graphs of their mathematical results while demonstrating reasoning in the context of the given event, observation, or phenomenon.

SP 6: Constructing Explanations and Engaging in Argument from Evidence

- ❑ Instruction presents an event, observation, or phenomenon.
- ❑ Instruction presents or asks instructor/students to make a claim based on the given event, observation, or phenomenon.
- ❑ Instruction has instructor/students provide scientific principles or evidence (data or observations) to support the claim.
- ❑ Instruction has instructor/students provide reasoning about why the scientific principles or evidence support the claim.

SP 7: Evaluating Information

- ❑ Instruction provides an excerpt from a conversation, article, student solution, or other communication that makes one or more assertions.
- ❑ Instruction presents a conclusion about the validity of the assertion(s) made or asks instructor/students to make a conclusion about the validity of the assertion(s), or reconcile multiple assertions with each other.
- ❑ Instruction has instructor/students provide reasoning to support their conclusion(s) about the validity of the assertion(s) or reconciliation with data, observations, or scientific principles.

Causal Mechanistic Reasoning

How:

- Collisions between molecules lead to forward and reverse reactions occurring.
- Changes in concentration or temp alter the relative rate of success of collisions to lead to the forward or reverse reaction. (Level below the process of interest)

Why:

- Changes in concentration alter the relative forward and reverse reaction rates by increasing number of reactant collisions which increases the number of successful collisions
- Changes in temperature result in more collisions that can successfully overcome the activation energy barrier. Due to the differences in the activation energy barriers for the forward and reverse reactions, the two processes will be differentially impacted.
- If rates are unequal, then the rate of one will increase and the other decrease (due to changing concentrations of reactants and products) until they become equal.

Must have both parts though can explain only concentration (bullet 1 of Why) or only temp (bullet 2 of Why)!

Multimedia Principles

Multimedia learning as knowledge construction: The goal of multimedia presentations is not only to present information but also to provide guidance for how to process the presented information – that is, for determining what to pay attention to, how to mentally organize it, and how to relate it to prior knowledge.

We will categorize the videos using each of the following criteria (High and Low should be mutually exclusive.)

High	Low
● Video almost exclusively involves **meaningful and relevant** images and verbal components AND **images are explained for a novice learner**	● Large segments of video presented just verbally and not pictorially (ex. just the instructor talking, or just written words) OR images presented are not relevant to learning or explained. **If images are clearly incorrect or misleading it should be low.**
● Text on screen is minimal (ex. Only brief bullet points or keywords if text is used)	● Contains full sentences of written text that are also narrated (e.g., several bullet points or sentences on one screen OR full sentences of text on many screens)
● Content is all relevant to the topic and learning (no music, unhelpful animations, graphics, etc.)	● Contains extraneous content, music, graphics, animations, background changes, sounds, or just too much stuff on the screen at one time - **anything that detracts attention from core content**
● Contains elements that **meaningfully** support student organizing content or identify key ideas (introductory organizer, section heading, summary, guiding questions/topic (for short, focused video only) **Key Question: Could a novice learner clearly understand what they should take away from the video? Yes=high.**	● Does not contain any elements that meaningfully help learners organize content or identify key ideas (e.g., no introductory organizer, section headings, video segments, summary, guiding question/topic (for short focused videos only)

Video Exemplars

Below are specific examples to help the reader understand the criteria. Each example is taken from the set of LCP videos evaluated. In Table S1, we include a specific time frame within the video where the topic is demonstrated. However, there may be multiple examples within the video, but we have only indicated one specific incidence for simplicity.

Table S1: Examples of videos meeting specific characteristics

Criteria	Channel	Link	Time in video
Video making clear connections between macroscopic and symbolic levels	Melissa Rathier	https://youtu.be/RjFW3smI1fY?si=fylqfHszMBbnbtDA&t=15	0:15-1:41
Video using core idea to explain LCP	CrashCourse	https://youtu.be/g5wNg_dKsYY?si=LYb_JZSXdxF03Tt7&t=300	5:00-5:30
Video including a science practice	Bozeman Science	https://youtu.be/PciV_Wuh9V8?si=Os1ITCndD0NA1y5A&t=139	2:19-3:48
Video encouraging student self-assessment	Freesciencelessons	https://youtu.be/utmV4Q0t6MI?si=gXrMRWuKm46YunUG&t=129	2:09-2:35
Video using an analogy both verbally and visually (equilibrium being like an escalator)	Extraclass Official	https://youtu.be/iiO2PkL9jsg?si=H4XMuCh43lMYtONc&t=11	0:11
Video using a potentially misleading balance to explain equilibrium	CrashCourse	https://youtu.be/g5wNg_dKsYY?si=LYb_JZSXdxF03Tt7&t=300	5:00-5:30
Video with anthropomorphizing the chemical reactions and chemicals	Science Simplified	https://youtu.be/KoHUbyWAplQ?si=GxXPkWqK8aOHXMzM&t=11	0:11-0:23

Checklist for video evaluation or creation

The checklist below is provided to help an individual seeking to the described framework to either evaluate an existing video or to help in the planning when creating a video on a specific chemistry topic. For video production suggestions, see Castillo, et al. (2021).

Johnstone's Triangle Elements

Identify which of the following relevant representations are present within the video. Suitable representations will need to be defined by the user as they may be content topic dependent.

☐ Macroscopic

☐ Particulate

☐ Symbolic

☐ Were the different levels explicitly linked together

Core Ideas

Identify if a core idea was present within the video. Core ideas must be defined by the user as they are highly content topic dependent. This framework was developed around the chemistry core ideas outlined in the 3D-LAP (Laverty, et al, 2016) or adapted from the Framework (NRC, 2021). Ideally, a core idea is woven through the full explanation of a concept.

☐ Present ☐ Not Present

Scientific Practices

Identify if a science practice was present within a video. The criteria for science practices have been explicitly defined in the 3D-LOP (Bain, et al., 2020) and are found on page 4 of this document. Ideally the science practice is connected to the core idea as recommended in the Framework (NRC, 2021).

☐ Present ☐ Not Present

Causal Mechanistic Reasoning

Identify if causal mechanistic reasoning (CMR) idea was present within the video. CMR must be defined by the user as it is highly content topic dependent. CMR mush have a clearly defined question (what) that it is answering. It must both provide a how it happens (cause) and a scientifically valid reasoning (mechanism) for why it happens. The mechanism will be at a level below the phenomenon itself. The core "what" question, is typically related to the core ideas for the concept.

☐ Present ☐ Not Present

Self-assessment

Does the video specifically encourage self-assessment by the viewer? This typically involves posing a question and then providing the viewer with a specific prompt to pause the video to attempt to assess their own knowledge. The video should then reveal the answer either explicitly or by providing data (often in the form of a demonstration) that would allow the viewer to understand if their assessment was correct.

☐ Present ☐ Not Present

Analogies

Does the video employ analogies? If so, are they presented visually (so that a viewer who may be unfamiliar with the analogies may still benefit from) or only verbally only (requiring the viewer to have some prior knowledge).

☐ Present verbally and visually ☐ Present, verbally only ☐ Not Present

Captions

Does the video rely exclusively on auto-captioning? If so, the video may be lacking punctuation and capitalization. It is also more likely to have misspelled scientific words.

☐ Auto captioned ☐ Fully captioned

Multimedia Principles

Following good multimedia principles for creating educational materials can help strongly support learning. Identify which statement best categorizes a video.

**High**	**Low**
**Image usage**	
• Video almost exclusively involves **meaningful and relevant** images and verbal components AND **images are explained for a novice learner**<br><br>☐ High	• Large segments of video presented just verbally and not pictorially (ex. just the instructor talking, or just written words) OR images presented are not relevant to learning or explained. **If images are clearly incorrect or misleading it should be low.**<br><br>☐ Low

Text usage	
• Text on screen is minimal (ex. Only brief bullet points or keywords if text is used)<br><br>☐ High	• Contains full sentences of written text that are also narrated (e.g., several bullet points or sentences on one screen OR full sentences of text on many screens)<br><br>☐ Low
**Video Coherence**	
• Content is all relevant to the topic and learning (no music, unhelpful animations, graphics, etc.)<br><br>☐ High	• Contains extraneous content, music, graphics, animations, background changes, sounds, or just too much stuff on the screen at one time - **anything that detracts attention from core content**<br><br>☐ Low
**Video Segmenting and Supports**	
• Contains elements that **meaningfully** support student organizing content or identify key ideas (introductory organizer, section heading, summary, guiding questions/topic (for short, focused video only) **Key Question: Could a novice learner clearly understand what they should take away from the video? Yes=high.**<br><br>☐ High	Does not contain any elements that meaningfully help learners organize content or identify key ideas (e.g., no introductory organizer, section headings, video segments, summary, guiding question/topic (for short focused videos only)<br><br>☐ Low

References:

Bain, K.; Bender, L.; Bergeron, P.; Caballero, M. D.; Carmel, J. H.; Duffy, E. M.; Ebert-May, D.; Fata-Hartley, C. L.; Herrington, D. G.; Laverty, J. T.; Matz, R. L.; Nelson, P. C.; Posey, L. A.; Stoltzfus, J. R.; Stowe, R. L.; Sweeder, R. D.; Tessmer, S. H.; Underwood, S. M.; Urban-Lurain, M.; Cooper, M. M. Characterizing College Science Instruction: The Three-Dimensional Learning Observation Protocol. *PLOS ONE* **2020**, *15* (6), e0234640. https://doi.org/10.1371/journal.pone.0234640.

Castillo, S.; Calvitti, K.; Shoup, J.; Rice, M.; Lubbock, H,; Oliver, K.H. Production Processes for Creating Educational Videos. *CBE Life Sciences Education* **2021**, 20 (2), 10:es71-12. https://doi.org/10.1187/cbe.20-06-0120.

Laverty, J. T.; Underwood, S. M.; Matz, R. L.; Posey, L. A.; Carmel, J. H.; Caballero, M. D.; Fata-Hartley, C. L.; Ebert-May, D.; Jardeleza, S. E.; Cooper, M. M. Characterizing College Science Assessments: The Three-Dimensional Learning Assessment Protocol. *PLoS ONE* **2016**, *11*, e0162333. https://doi.org/10.1371/journal.pone.0162333.

National Research Council. *A Framework for K-12 Science Education: Practices, Crosscutting Concepts, and Core Ideas*; The National Academies Press: Washington, DC, 2012.
